# C—H⋯O contacts in the crystal structure of 1,3-di­thiane 1,1,3,3-tetra­oxide

**DOI:** 10.1107/S2056989021000876

**Published:** 2021-01-29

**Authors:** Richard L. Harlow, Allen G. Oliver, Michael P. Sammes

**Affiliations:** a 624 Erlen Rd., Plymouth Meeting, PA 19462 , USA; b Dept. of Chemistry and Biochemistry, University of Notre Dame, Notre Dame, IN 46556-5670, USA; c 2 Baydons Lane, Chippenham SN15 3JX, UK

**Keywords:** crystal structure, 1,3-di­thiane 1,1,3,3,-tetra­oxide, C—H⋯O contacts

## Abstract

The crystal structure of 1,3-di­thiane 1,1,3,3-tetra­oxide, C_4_H_8_O_4_S_2_, has been determined to examine the inter­molecular C—H⋯O hydrogen bonds in a small mol­ecule with highly polarized hydrogen atoms. The crystals are monoclinic, space group *P*n, with *a* = 4.9472 (5), *b* = 9.9021 (10), *c* = 7.1002 (7) Å and β = 91.464 (3)° with *Z* = 2. The mol­ecules form two stacks parallel to the *a* axis with the molecules being one *a* translation distance from each other. This stacking involves axial hydrogen atoms on one mol­ecule and the axial oxygen atoms on the adjacent mol­ecule in the stack. None of these C—H⋯O contacts is particularly short (all are > 2.4 Å). The many C—H⋯O contacts between the two stacks involve at least one equatorial hydrogen or oxygen atom. Again, no unusually short contacts are found. The whole crystal structure basically consists of a complex network of C—H⋯O contacts with no single, linear C—H⋯O contacts, only contacts that involve two (bifurcated), and mostly three or four neighbors.

## Abstract 2

In the title compound, the mol­ecules stack parallel with the *a* axis with multiple H⋯O contacts involving the axial H and O atoms. Many more H⋯O contacts between the stacks exist, which mostly involve the equatorial hydrogen and oxygen atoms. The highly polarized hydrogen atoms of the –SO_2_—CH_2_—SO_2_– moiety make no exceptionally short H⋯O contacts but clearly play a leading role in the formation of the stacks.

## Chemical context   

This is the second in a series looking at the C—H⋯O contacts in cyclic organic structures containing multiple sulfone groups [see Harlow *et al.* (2019 ▸) for the first structure in the series: 1,4-di­thiane 1,1,4,4-tetra­oxide]. Any methyl­ene group adjacent to a sulfone has polarized hydrogen atoms. Methyl­ene groups bonded to two sulfones are polarized to such an extent that they may form a C—H⋯*X* (*X* = N, O) hydrogen bond (Harlow *et al.*, 1984 ▸) as illustrated.
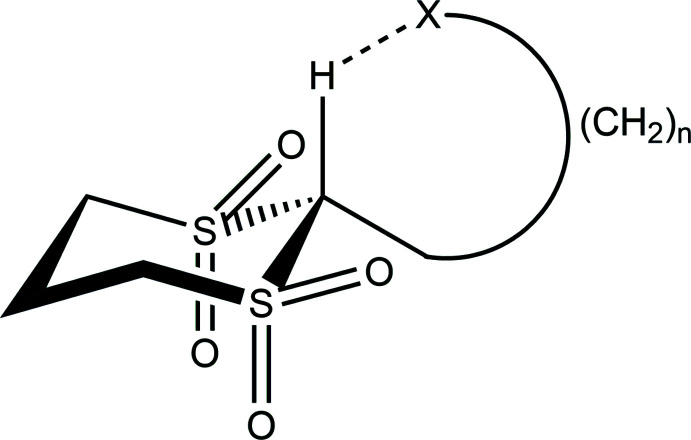


The structure of the unsubstituted 1,3-di­sulfone, however, has not been previously reported and was of inter­est particularly because of its high melting point (583 K) and decomposition temperatures (*ca* 623 K), which are suggestive of potentially strong C—H⋯O contacts. The ^1^H NMR spectrum of the compound dissolved in DMSO shows a singlet, 2H, at δ = 5.238 ppm (highly polarized hydrogen atoms on C2 between the two SO_2_ groups); a triplet, 4H, at 3.370 ppm (moderately polarized hydrogen atoms on C4 and C6 with one adjacent SO_2_ group); and a pentet, 2H, at 2.260 ppm (relatively unpolarized hydrogen atoms on C5). See Li & Sammes (1983 ▸) for further details of ^1^H NMR spectra and hydrogen-atom polarity in di­sulfones.
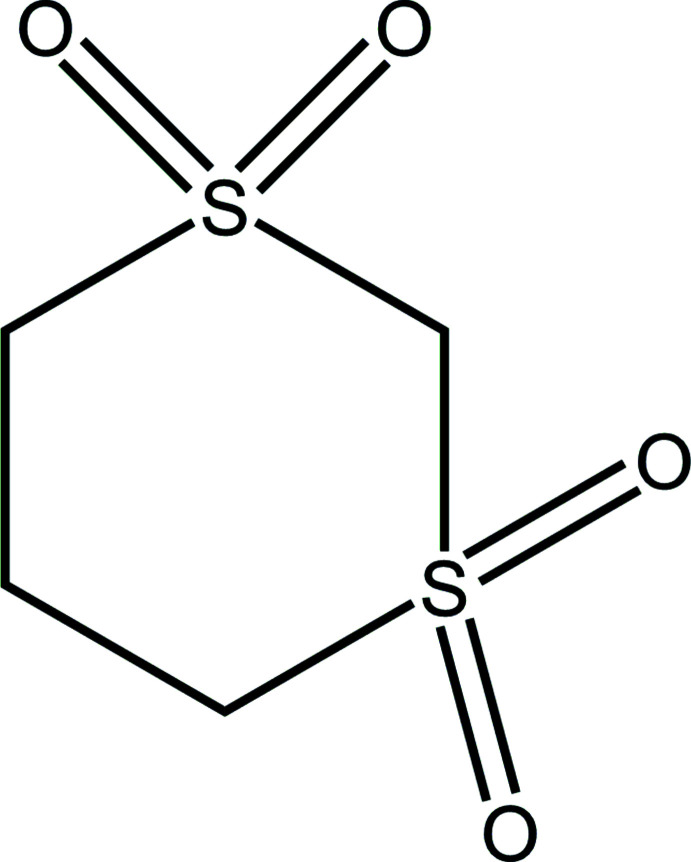


## Structural commentary   

**Figure 1:**
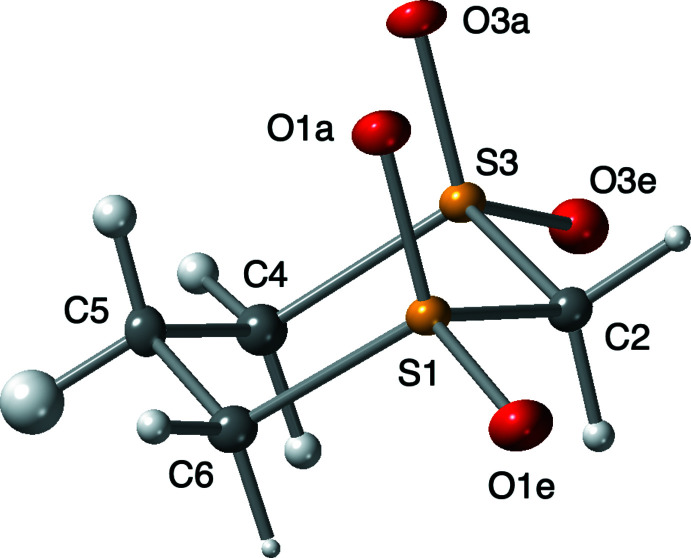
Labeled *ORTEP* drawing (50% probability) of the 1,3-di­thiane 1,1,3,3-tetra­oxide mol­ecule. The lower case suffixes ‘a′ and ‘e′ are used to distinguish whether the atoms are in the axial or equatorial position on the ring.

Fig. 1 ▸ is an ORTEP drawing of the 1,3-di­thiane 1,1,3,3-tetra­oxide mol­ecule with atom labels using the suffix ‘a′ for axial and ‘e′ for equatorial atoms. The bond distances and angles are very similar to those reported for the 1,4-di­sulfone taking into consideration the small amount of distortion related to the different positions of the two sulfonyl groups, *i.e*. 1,3- *vs* 1,4-sites in the ring.

The hydrogen atoms were fully refined with isotropic displacement parameters as there seemed to be a correlation between the ‘strength’ of the C—H polarization and the displacement parameters of the hydrogen atoms: more polarization seems to yield a smaller radius. Any further comments on this correlation from a structural standpoint would require a diffraction study using neutrons instead of X-rays to better define both the positions and the displacement parameters of the hydrogen atoms.

## Supra­molecular features   

**Figure 2:**
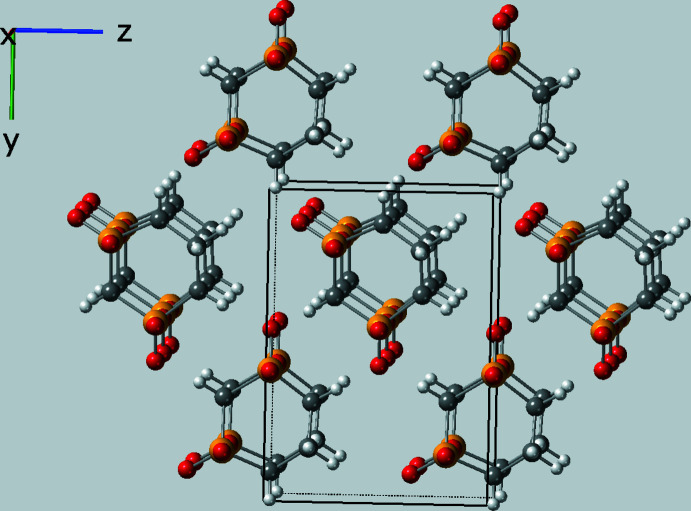
Packing diagram showing the stacking of the mol­ecules parallel to the *a* axis.

**Figure 3:**
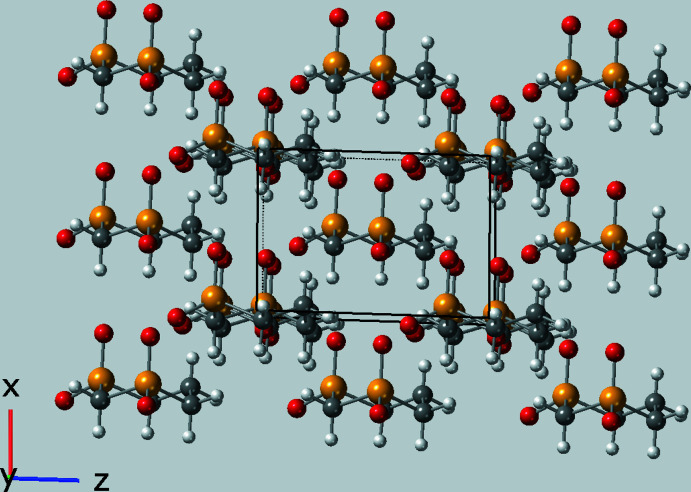
Packing diagram rotated to a view approximately parallel to the *b* axis to show that the stacks created by the *n*-glide are displaced by *x* + 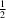. The figure also shows that both stacks are crystallographically polar with, for example, all the axial oxygen atoms pointing upward and most of the axial hydrogen atoms pointing downward except those on C5.

**Figure 4:**
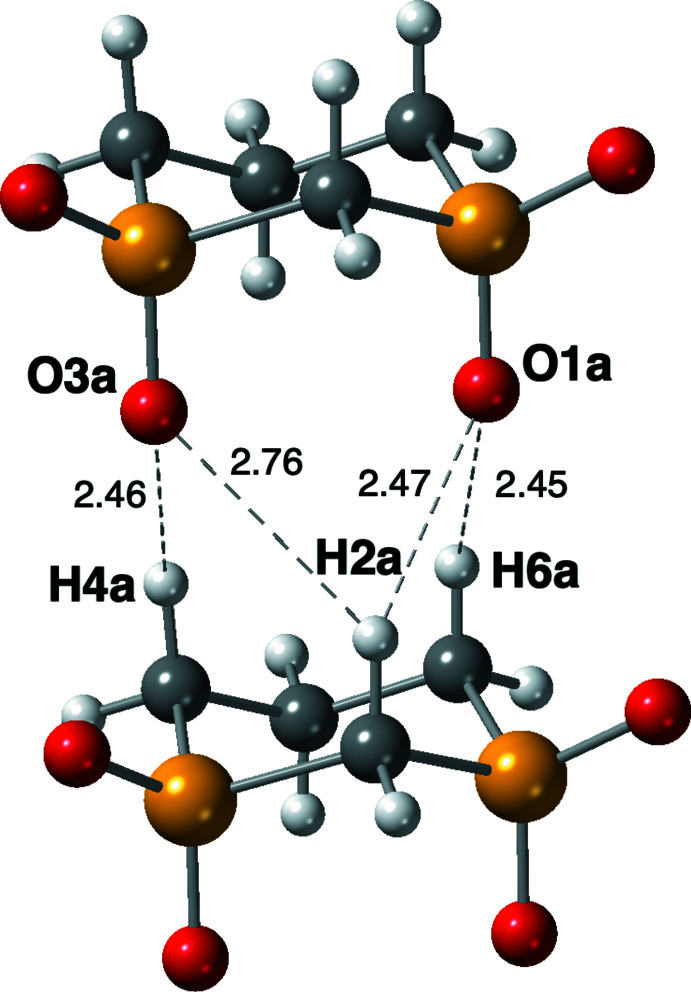
Two adjacent mol­ecules of a stack with the O⋯H contacts detailed. The discrepancy in the H2*a*⋯O bonds is caused by the mol­ecules in the stacks being slightly tilted as evidenced by S1 and S3 not having the same *x* coordinate: 0.568 *vs* 0.546 (even though the mol­ecules are related by a translation along *a*). This leads to a difference in the S1⋯S3^i^ and S3⋯S1^i^ distances, for example, of 5.646 *vs* 5.898 Å. The longer S⋯S distance is associated with the longer H2*a*⋯O distance.

Obviously, it is the packing of the mol­ecules that is especially fascinating given that the mol­ecules stack parallel to the *a* axis at a distance of one translation on *a*, *i.e.* the length of the *a* axis, *ca* 4.9472 (5) Å at 120 K. A general packing diagram is shown in Fig. 2 ▸. The *n*-glide, the only symmetry operator in space group *Pn*, curiously preserves the polarity of the stacks and creates a polar crystal with, for example, all of the axial oxygen atoms in the stacks pointing in the same *a*-axis direction (up in Fig. 3 ▸) and most of the axial hydrogen atoms pointing down. The stacking is directly stabilized by C—H⋯O contacts between neighboring mol­ecules in the stack and only involves the axial oxygen and hydrogen atoms (see Fig. 4 ▸ for the details). In Table 1 ▸, these axial C—H⋯O contacts are designated with symmetry ‘i′.

In addition, there are multiple O⋯H contacts between the stacks, all of which involve at least one equatorial atom. Some of these also serve to bridge adjacent mol­ecules within a stack further cementing the mol­ecules in the stack. Examination of the O⋯H contacts in Table 1 ▸ quickly shows that there are no short H⋯O contacts (disappointing) but simply a plethora of contacts that hold the mol­ecules in this crystal structure together. When the environment of each oxygen atom is surveyed in detail, it is found that they all inter­act with four hydrogen atoms, which generally form a distorted quadrilateral with O⋯H contact distances that vary from 2.44 to 3.09 Å. This is very similar to what was found for the 1,4-di­sulfone structure where example figures can be found (Harlow *et al.*, 2019 ▸). The main difference is that the O⋯H distances in the 1,4-di­sulfone structure were relatively uniform and, in this structure, they are not.

One mystery that remains is why a small mol­ecule with mirror symmetry crystallizes in a non-centrosymmetric, polar space group?

## Database survey   

A Cambridge Crystallographic Database survey of the 1,3-di­sulfone moiety reveals 22 structures with that motif (CSD version 5.41 + three updates; Groom *et al.*, 2016 ▸). Four of the structures were authored by Harlow and Sammes and served as an impetus for the present study. A paper entitled in part ‘Study of the Inter­action of Silver(I) with β-Di­sulfone in Aqueous Alkaline Media’ was of particular inter­est because it suggested that metal salts could be made with our title compound, *i.e*. one of the hydrogen atoms on C2 was acidic enough to be easily removed (DeMember *et al.*, 1983 ▸) in a solution of KOH.

## Synthesis and crystallization   

To a stirred solution of 1,3-di­thiane (1.05 g, 5.70 mmol, Alfa-Aeser) in glacial acetic acid (25 mL) was added a solution of 30% H_2_O_2_ (10 mL) in glacial acetic acid (25 mL) and the mixture was heated to 323 K overnight. The white precipitate was separated on a Buchner funnel and washed with water (3 × 25 mL) and dried in air. Colorless, rod-like crystals of the compound were harvested from an evaporated KOH (0.5 *M*) solution of the the 1,3-di­sulfone. ^1^H NMR (400 MHz, DMSO-*d*_6_), δ: 5.238 (*singlet*, 2H, H2*a*/e), 3.370 (*triplet*, 4H, ^3^ *J*_H,H_ = 5 Hz, H4*a*/e, H6*a*/e), 2.260 (*pentet*, 2H, ^3^ *J*_H,H_ = 6 Hz, H5*a*/e); ^13^C NMR (100.13 MHz, DMSO-*d*_6_), δ: 70.12 (C2), 50.09 (C4/C6), 17.60 (C4). HRMS (negative ion mode, [C_4_H_7_O_4_S_2_]^−^) *m*/*z* found: 182.9803; calculated: 182.9786.

## Refinement   

Crystal data, data collection and structure refinement details are summarized in Table 2 ▸. Non-hydrogen atoms were refined with anisotropic displacement parameters and all hydrogen atoms were located from a difference-Fourier map and refined freely.

## Supplementary Material

Crystal structure: contains datablock(s) I. DOI: 10.1107/S2056989021000876/ey2004sup1.cif

Structure factors: contains datablock(s) I. DOI: 10.1107/S2056989021000876/ey2004Isup2.hkl

CCDC reference: 2058562

Additional supporting information:  crystallographic information; 3D view; checkCIF report

## Figures and Tables

**Table 1:** Hydrogen-bond geometry (Å, °)

*D*—H⋯*A*	*D*—H	H⋯*A*	*D*⋯*A*	*D*—H⋯*A*
C2—H2*a*⋯O1*a* ^i^	0.98 (4)	2.47 (4)	3.315 (4)	143 (3)
C2—H2*a*⋯O3*a* ^i^	0.98 (4)	2.76 (4)	3.520 (5)	134 (3)
C2—H2*a*⋯O3*e* ^ii^	0.98 (4)	2.91 (4)	3.556 (4)	124 (3)
C2—H2*e*⋯O3*a* ^ii^	0.97 (4)	2.49 (4)	3.201 (4)	130 (3)
C2—H2*e*⋯O3*e* ^iii^	0.97 (4)	2.49 (4)	3.370 (4)	151 (3)
C4—H4*a*⋯O3*a* ^i^	0.99 (4)	2.46 (4)	3.329 (4)	147 (3)
C4—H4*e*⋯O3*a* ^iv^	0.94 (4)	2.70 (4)	3.462 (4)	138 (3)
C4—H4*e*⋯O3*e* ^v^	0.94 (4)	2.63 (4)	3.454 (5)	146 (3)
C5—H5*e*⋯O1*a* ^vi^	1.00 (5)	2.91 (5)	3.598 (5)	127 (3)
C5—H5*e*⋯O1*e* ^vii^	1.00 (5)	2.50 (5)	3.395 (4)	150 (4)
C6—H6*a*⋯O1*a* ^i^	0.95 (4)	2.45 (4)	3.287 (4)	147 (3)
C6—H6*a*⋯O1*e* ^vi^	0.95 (4)	2.65 (4)	3.269 (5)	124 (3)
C6—H6*e*⋯O1*a* ^vi^	0.95 (4)	2.81 (4)	3.344 (5)	116 (3)
C6—H6*e*⋯O1*e* ^viii^	0.95 (4)	2.44 (4)	3.186 (5)	135 (3)

Symmetry codes: (i) 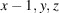; (ii) 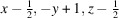; (iii) 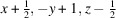; (iv) 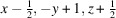; (v) 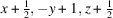; (vi) 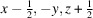; (vii) 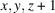; (viii) 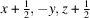.

**Table 2:** Experimental details

Crystal data	
Chemical formula	C_4_H_8_O_4_S_2_
*M*_r_	184.22
Crystal system, space group	Monoclinic, *P* *n*
Temperature (K)	120
*a*, *b*, *c* (Å)	4.9472 (5), 9.9021 (10), 7.1002 (7)
β (°)	91.464 (3)
*V* (Å^3^)	347.71 (6)
*Z*	2
Radiation type	Mo *K*α
μ (mm^−1^)	0.72
Crystal size (mm)	0.54 × 0.11 × 0.05

Data collection	
Diffractometer	Bruker PHOTON-II
Absorption correction	Multi-scan (*SADABS*; Krause *et al.*, 2015 ▸)
*T*_min_, *T*_max_	0.579, 0.746
No. of measured, independent and observed [*I* > 2σ(*I*)] reflections	6109, 1513, 1473
*R*_int_	0.039
(sin θ/λ)_max_ (Å^−1^)	0.640

Refinement	
*R*[*F* ^2^ > 2σ(*F* ^2^)], *wR*(*F* ^2^), *S*	0.027, 0.055, 1.15
No. of reflections	1513
No. of parameters	123
No. of restraints	2
H-atom treatment	All H-atom parameters refined
Δρ_max_, Δρ_min_ (e Å^−3^)	0.28, −0.40
Absolute structure	Flack *x* determined using 682 quotients [(*I* ^+^)−(*I* ^−^)]/[(*I* ^+^)+(*I* ^−^)] (Parsons *et al.*, 2013 ▸).
Absolute structure parameter	0.07 (4)

Computer programs: *APEX3* and *SAINT* (Bruker, 2015 ▸), *olex2.solve* (Dolomanov *et al.*, 2009 ▸), *SHELXL2018/3* (Sheldrick, 2015 ▸), *CrystalMaker* (Palmer, 2014 ▸), and *publCIF* (Westrip, 2010 ▸).

## supplementary crystallographic information

### Crystal data

**Table:**

C_4_H_8_O_4_S_2_	*F*(000) = 192
*M**_r_* = 184.22	*D*_x_ = 1.760 Mg m^−^^3^
Monoclinic, *P**n*	Mo *K*α radiation, λ = 0.71073 Å
*a* = 4.9472 (5) Å	Cell parameters from 5408 reflections
*b* = 9.9021 (10) Å	θ = 3.5–27.1°
*c* = 7.1002 (7) Å	µ = 0.72 mm^−^^1^
β = 91.464 (3)°	*T* = 120 K
*V* = 347.71 (6) Å^3^	Rod, colorless
*Z* = 2	0.54 × 0.11 × 0.05 mm

### Data collection

**Table:**

Bruker PHOTON-II diffractometer	1513 independent reflections
Radiation source: fine-focus sealed tube	1473 reflections with *I* > 2σ(*I*)
Bruker TRIUMPH curved-graphite monochromator	*R*_int_ = 0.039
Detector resolution: 7.41 pixels mm^-1^	θ_max_ = 27.1°, θ_min_ = 2.1°
combination of ω and φ–scans	*h* = −6→6
Absorption correction: multi-scan (SADABS; Krause *et al.*, 2015)	*k* = −12→12
*T*_min_ = 0.579, *T*_max_ = 0.746	*l* = −9→9
6109 measured reflections	

### Refinement

**Table:**

Refinement on *F*^2^	Secondary atom site location: difference Fourier map
Least-squares matrix: full	Hydrogen site location: difference Fourier map
*R*[*F*^2^ > 2σ(*F*^2^)] = 0.027	All H-atom parameters refined
*wR*(*F*^2^) = 0.055	*w* = 1/[σ^2^(*F*_o_^2^) + (0.0163*P*)^2^ + 0.148*P*] where *P* = (*F*_o_^2^ + 2*F*_c_^2^)/3
*S* = 1.15	(Δ/σ)_max_ < 0.001
1513 reflections	Δρ_max_ = 0.28 e Å^−^^3^
123 parameters	Δρ_min_ = −0.40 e Å^−^^3^
2 restraints	Absolute structure: Flack *x* determined using 682 quotients [(*I*^+^)-(*I*^-^)]/[(*I*^+^)+(*I*^-^)] (Parsons *et al*., 2013).
Primary atom site location: iterative	Absolute structure parameter: 0.07 (4)

### Special details

**Table:**

Geometry. All esds (except the esd in the dihedral angle between two l.s. planes) are estimated using the full covariance matrix. The cell esds are taken into account individually in the estimation of esds in distances, angles and torsion angles; correlations between esds in cell parameters are only used when they are defined by crystal symmetry. An approximate (isotropic) treatment of cell esds is used for estimating esds involving l.s. planes.

### Fractional atomic coordinates and isotropic or equivalent isotropic displacement parameters (Å^2^)

**Table:**

	*x*	*y*	*z*	*U*_iso_*/*U*_eq_	
S1	0.56801 (14)	0.16084 (8)	0.31351 (11)	0.01036 (19)	
S3	0.54595 (15)	0.42138 (8)	0.52142 (12)	0.01083 (19)	
O1a	0.8592 (5)	0.1643 (3)	0.3226 (4)	0.0167 (5)	
O1e	0.4403 (5)	0.0944 (3)	0.1549 (4)	0.0173 (6)	
O3a	0.8376 (5)	0.4249 (3)	0.5291 (4)	0.0162 (5)	
O3e	0.4048 (5)	0.5484 (2)	0.5189 (4)	0.0170 (6)	
C2	0.4419 (7)	0.3292 (4)	0.3144 (5)	0.0125 (7)	
C4	0.4299 (7)	0.3185 (4)	0.7058 (5)	0.0155 (7)	
C5	0.5438 (8)	0.1749 (4)	0.6975 (5)	0.0166 (8)	
C6	0.4464 (8)	0.0935 (4)	0.5261 (5)	0.0155 (8)	
H2a	0.244 (8)	0.323 (3)	0.309 (6)	0.006 (9)*	
H2e	0.515 (8)	0.376 (4)	0.206 (6)	0.004 (9)*	
H4a	0.231 (8)	0.326 (4)	0.697 (5)	0.007 (9)*	
H4e	0.489 (7)	0.362 (4)	0.817 (6)	0.011 (10)*	
H5a	0.724 (9)	0.179 (4)	0.706 (6)	0.011 (10)*	
H5e	0.475 (9)	0.123 (5)	0.807 (7)	0.025 (12)*	
H6a	0.255 (7)	0.094 (4)	0.513 (6)	0.002 (8)*	
H6e	0.523 (8)	0.005 (4)	0.529 (6)	0.007 (8)*	

### Atomic displacement parameters (Å^2^)

**Table:**

	*U*^11^	*U*^22^	*U*^33^	*U*^12^	*U*^13^	*U*^23^
S1	0.0083 (4)	0.0114 (4)	0.0114 (4)	0.0005 (4)	0.0006 (3)	−0.0008 (3)
S3	0.0098 (4)	0.0107 (4)	0.0120 (4)	−0.0004 (3)	−0.0004 (3)	−0.0015 (3)
O1a	0.0091 (12)	0.0200 (13)	0.0209 (14)	0.0015 (10)	0.0017 (10)	−0.0001 (11)
O1e	0.0161 (13)	0.0203 (13)	0.0153 (13)	−0.0022 (11)	−0.0004 (10)	−0.0076 (10)
O3a	0.0085 (12)	0.0191 (13)	0.0210 (13)	−0.0026 (10)	−0.0019 (10)	−0.0017 (11)
O3e	0.0167 (14)	0.0120 (13)	0.0224 (14)	0.0027 (10)	0.0024 (11)	−0.0019 (10)
C2	0.0120 (18)	0.0124 (17)	0.0130 (17)	−0.0006 (13)	−0.0015 (14)	0.0003 (13)
C4	0.0146 (18)	0.0211 (19)	0.0108 (16)	0.0000 (15)	0.0023 (14)	−0.0010 (14)
C5	0.0138 (19)	0.022 (2)	0.0136 (19)	0.0005 (15)	0.0007 (15)	0.0071 (14)
C6	0.0119 (16)	0.0177 (19)	0.0170 (18)	0.0002 (14)	−0.0002 (14)	0.0021 (14)

### Geometric parameters (Å, º)

**Table:**

S1—O1e	1.437 (3)	C2—H2e	0.97 (4)
S1—O1a	1.441 (3)	C4—C5	1.531 (5)
S1—C6	1.769 (4)	C4—H4a	0.99 (4)
S1—C2	1.780 (4)	C4—H4e	0.94 (4)
S3—O3e	1.439 (3)	C5—C6	1.528 (5)
S3—O3a	1.443 (3)	C5—H5a	0.89 (4)
S3—C4	1.767 (4)	C5—H5e	1.00 (5)
S3—C2	1.795 (4)	C6—H6a	0.95 (4)
C2—H2a	0.98 (4)	C6—H6e	0.95 (4)
			
O1e—S1—O1a	117.72 (16)	C5—C4—S3	112.3 (3)
O1e—S1—C6	110.10 (17)	C5—C4—H4a	116 (2)
O1a—S1—C6	109.38 (17)	S3—C4—H4a	105 (2)
O1e—S1—C2	106.54 (16)	C5—C4—H4e	111 (2)
O1a—S1—C2	109.14 (16)	S3—C4—H4e	105 (3)
C6—S1—C2	102.89 (17)	H4a—C4—H4e	108 (3)
O3e—S3—O3a	117.64 (15)	C6—C5—C4	114.3 (3)
O3e—S3—C4	110.27 (17)	C6—C5—H5a	112 (3)
O3a—S3—C4	109.24 (17)	C4—C5—H5a	109 (3)
O3e—S3—C2	107.81 (16)	C6—C5—H5e	104 (3)
O3a—S3—C2	107.99 (16)	C4—C5—H5e	108 (3)
C4—S3—C2	102.81 (18)	H5a—C5—H5e	109 (4)
S1—C2—S3	112.73 (19)	C5—C6—S1	111.9 (3)
S1—C2—H2a	107 (2)	C5—C6—H6a	112 (2)
S3—C2—H2a	109 (2)	S1—C6—H6a	106 (2)
S1—C2—H2e	108 (2)	C5—C6—H6e	111 (3)
S3—C2—H2e	107 (2)	S1—C6—H6e	103 (2)
H2a—C2—H2e	113 (3)	H6a—C6—H6e	114 (3)
			
O1e—S1—C2—S3	172.32 (18)	O3a—S3—C4—C5	−57.4 (3)
O1a—S1—C2—S3	−59.6 (2)	C2—S3—C4—C5	57.1 (3)
C6—S1—C2—S3	56.5 (2)	S3—C4—C5—C6	−66.1 (4)
O3e—S3—C2—S1	−172.54 (18)	C4—C5—C6—S1	66.5 (4)
O3a—S3—C2—S1	59.4 (2)	O1e—S1—C6—C5	−171.4 (3)
C4—S3—C2—S1	−56.0 (2)	O1a—S1—C6—C5	57.8 (3)
O3e—S3—C4—C5	171.8 (3)	C2—S1—C6—C5	−58.2 (3)

### Hydrogen-bond geometry (Å, º)

**Table:**

*D*—H···*A*	*D*—H	H···*A*	*D*···*A*	*D*—H···*A*
C2—H2*a*···O1*a*^i^	0.98 (4)	2.47 (4)	3.315 (4)	143 (3)
C2—H2*a*···O3*a*^i^	0.98 (4)	2.76 (4)	3.520 (5)	134 (3)
C2—H2*a*···O3*e*^ii^	0.98 (4)	2.91 (4)	3.556 (4)	124 (3)
C2—H2*e*···O3*a*^ii^	0.97 (4)	2.49 (4)	3.201 (4)	130 (3)
C2—H2*e*···O3*e*^iii^	0.97 (4)	2.49 (4)	3.370 (4)	151 (3)
C4—H4*a*···O3*a*^i^	0.99 (4)	2.46 (4)	3.329 (4)	147 (3)
C4—H4*e*···O3*a*^iv^	0.94 (4)	2.70 (4)	3.462 (4)	138 (3)
C4—H4*e*···O3*e*^v^	0.94 (4)	2.63 (4)	3.454 (5)	146 (3)
C5—H5*e*···O1*a*^vi^	1.00 (5)	2.91 (5)	3.598 (5)	127 (3)
C5—H5*e*···O1*e*^vii^	1.00 (5)	2.50 (5)	3.395 (4)	150 (4)
C6—H6*a*···O1*a*^i^	0.95 (4)	2.45 (4)	3.287 (4)	147 (3)
C6—H6*a*···O1*e*^vi^	0.95 (4)	2.65 (4)	3.269 (5)	124 (3)
C6—H6*e*···O1*a*^vi^	0.95 (4)	2.81 (4)	3.344 (5)	116 (3)
C6—H6*e*···O1*e*^viii^	0.95 (4)	2.44 (4)	3.186 (5)	135 (3)

Symmetry codes: (i) *x*−1, *y*, *z*; (ii) *x*−1/2, −*y*+1, *z*−1/2; (iii) *x*+1/2, −*y*+1, *z*−1/2; (iv) *x*−1/2, −*y*+1, *z*+1/2; (v) *x*+1/2, −*y*+1, *z*+1/2; (vi) *x*−1/2, −*y*, *z*+1/2; (vii) *x*, *y*, *z*+1; (viii) *x*+1/2, −*y*, *z*+1/2.
